# Functional response of *Anystis baccarum* (Acari: Anystidae) preying on two raspberry pests: *Aphis idaei* (Hemiptera: Aphididae) and *Neotetranychus rubi* (Acari: Tetranychidae)

**DOI:** 10.1093/jee/toaf112

**Published:** 2025-06-13

**Authors:** Jiunn Luh Tan, Rostislav Zemek

**Affiliations:** Department of Zoology, Faculty of Science, University of South Bohemia, České Budějovice, Czech Republic; Biology Centre CAS, Institute of Entomology, České Budějovice, Czech Republic; Biology Centre CAS, Institute of Entomology, České Budějovice, Czech Republic

**Keywords:** biological control of pests, integrated pest management, natural enemies, predatory mites, whirligig mites

## Abstract

Raspberry is an increasingly economically important soft fruit worldwide. To adopt the approaching EU Green Deal, growers are required to seek alternative pest management strategies. The predatory mite, *Anystis baccarum* (L.), which was recently discovered in raspberry, could be a promising candidate. However, the biology and predation capacity of this species in raspberry are unknown. This study aimed to investigate the functional response of *A. baccarum* to two common raspberry pests, *Aphis idaei* van der Goot and *Neotetranychus rubi* Trägårdh. In controlled laboratory conditions, six densities of *A. idaei* nymphs (2, 4, 8, 12, 16, and 24) and adult *N. rubi* females (2, 4, 8, 16, 24, and 32) were introduced in separate functional response experiments. Furthermore, the prey preference of *A. baccarum* on the two species was investigated when 5 *A. idaei* nymphs and adult *N. rubi* females were offered simultaneously to the predator. *Anystis baccarum* exhibited type II functional response to both prey, with capture rate for *A. idaei* higher than *N. rubi*. In addition, *A. idaei* was more likely to be consumed by *A. baccarum* than *N. rubi*. While promising as a biocontrol agent, the searching behavior, cannibalistic nature, and long generation time of *A. baccarum* suggest that it should not be relied upon solely for pest management in raspberry. Further studies on synergistic interactions with other biocontrol agents are recommended.

## Introduction

Anystidae, commonly known as whirligig mites, are a group of moderately large mites (0.5 to 1.5 mm) with long legs and are fast-moving. They have a long development time, with a full life cycle consisting of seven stages, namely, egg, prelarva, larva, protonymph, deutonymph, tritonymph, and adult stages (Meyer and Ueckermann 1987, Gerson et al. 2003, Zhang 2021). Anystids are generalist predators that feed on a wide range of insects and mites and are commonly found on both vegetation and the soil surface, where they hunt for food. In general, their searching behavior has been described as chance dependent, characterized by their random rapid zig-zag movement that allows them to encounter prey along their path (Meyer and Ueckermann 1987, Gerson et al. 2003, Saito et al. 2023). There are approximately 100 species in 20 genera of Anystidae described, among which *Anystis* is the better-known genus because several species are potential biocontrol agents against agricultural pests (Gerson et al. 2003, Zhang 2021). Among them, *Anystis baccarum* (L.) is gaining popularity because it is considered one of the potentially important natural enemies of pests in several crops, such as the European red spider mite, *Panonychus ulmi* (Koch), and the apple rust mite, *Aculus schlechtendali* (Nalepa), in apple orchards (Cuthbertson and Murchie 2004); *Tetranychus urticae* Koch, *P. ulmi*, leafhoppers, tarnished plant bug nymphs, and lepidopteran eggs in vineyards (Vincent and Lasnier 2020); English grain aphids, *Sitobion avenae* (Fabricius), and grain thrips, *Limothrips cerealium* (Haliday), in wheat fields (El Banhawy et al. 1993); and tea green leafhoppers, *Empoasca onukii* Matsuda, and smaller green leafhoppers, *Empoasca vitis* (Göthe), in tea plantations (Zeng et al. 2007, Chen et al. 2019). In addition, this species has been observed to prey on many economically important agricultural pests, such as whiteflies (ie *Bemisia tabaci* (Gennadius) and *Trialeurodes vaporariorum* (Westwood)), thrips (ie *Thrips tabaci* Lindeman*, Frankliniella occidentalis* (Pergande) and *Thrips parvispinus* (Karny)), aphids (ie *Myzus persicae* (Sulzer), *Macrosiphum euphorbiae* (Thomas), *Aulacorthum solani* (Kaltenbach), and *Rhopalosiphum padi* (L.)), and citrus mealybugs (*Planococcus citri* (Risso)) (Saito et al. 2023). *Anystis baccarum* was also found to be a potential biocontrol agent for augmentative biocontrol to manage populations of economically important pests in greenhouses, such as foxglove aphids, *A. solani*, and the western flower thrips, *F. occidentalis*. However, robust pest management is more achievable in the presence of other natural enemies, such as aphid parasitoids and phytoseiid predatory mites (Saito and Brownbridge 2021, 2022). In addition to living food, it also readily feeds on pollen (Saito and Brownbridge 2017), and possibly also exudates from extrafloral nectaries and sugar solutions (Gerson et al. 2003). In addition to having a wide dietary range, it is a cosmopolitan species, reported across several continents, including Australia, Europe, Africa, North and South America, and Asia (Meyer and Ueckermann 1987, Zeng et al. 2007, Cuthbertson and Murchie 2010, Chen et al. 2019, Saito and Brownbridge 2022). *Anystis baccarum* reproduces by thelytokous parthenogenesis, and thus far, it is believed that no males are present for this species (Meyer and Ueckermann 1987, Cuthbertson and Murchie 2010).

Despite the versatile nature of *A. baccarum*, as mentioned above, laboratory rearing of this mite, although possible (Golovach 1989), is challenging because of its cannibalistic behavior throughout its life cycle (Cuthbertson et al. 2014). Therefore, it was not introduced as augmentative biocontrol agents, but instead substantial effort has been made to promote the conservation of *A. baccarum* as a biocontrol agent in Northern Ireland apple orchards. (Cuthbertson et al. 2003, Cuthbertson 2004, Cuthbertson and Murchie 2005a, 2005b, 2010). However, more recent studies have demonstrated that laboratory rearing is possible using living plants, such as chrysanthemums and prey species, such as thrips and aphids, in an open-rearing system maintained within a walk-in growth chamber (Saito and Brownbridge 2017, 2021). Nevertheless, *A. baccarum* has been successfully commercialized in North America (Packer 2022). Despite these findings, more studies on *A. baccarum* are necessary, as its behavior may vary under different environmental conditions. For example, *Anystis* sp. has been reported to provide better control of spider mites on smooth compared to hairy leaf surfaces (Gerson 1992).

*Anystis baccarum* has recently been found in red raspberry in Norway and seems to contribute to the suppression of phytophagous mites in this crop (Tan et al. 2024b). Raspberry is an increasingly economically important soft fruit worldwide because of its rich nutritional content. Like other agricultural crops, raspberry can be damaged by several economically important pests (Tan et al. 2022), of which aphids, thrips, and phytophagous mites are known prey of *A. baccarum*. However, the abaxial surface of raspberry leaves is typically hairy. This raises curiosity about the performance of *A. baccarum* as a natural enemy of raspberry pests and whether it could be a candidate for biocontrol in raspberry orchards as an alternative raspberry pest management strategy to meet European (EU) Green Deal requirements in 2030. Therefore, this study aimed to investigate the predation behavior of *A. baccarum* toward two common raspberry pests, the small raspberry aphid *Aphis idaei* van der Goot and the spider mite *Neotetranychus rubi* Trägårdh. *Aphis idaei* is also a known vector of the raspberry vein chlorosis virus (RVCV, *Alphacytorhabdovirus alpharubi*) (Tan et al. 2022), and *N. rubi* is a potential vector of raspberry leaf blotch virus (RLBV, *Emaravirus idaeobati*) (Tan et al. 2024b), making effective management of these pests economically important. We evaluated the functional response of *A. baccarum*, as a key aspect of its predatory performance and potential for biological control. A functional response describes the change in per capita consumption of predators with changing prey density, considering factors such as capture rate, and handling time (Solomon 1949, Holling 1959, 1966). There are three types of functional responses: type I, where the predation rate increases linearly with prey density; type II, which follows a hyperbolic curve, with predation rate saturating at high prey abundance due to handling capacity limitations of the predator; and type III, which follows a sigmoid curve, where predation rate is low at low prey density, increases linearly at intermediate densities, and saturates at high densities (Holling 1959). Understanding the functional response exhibited by *A. baccarum* is an important step to evaluate its effectiveness in managing the target raspberry pests. In addition, the feeding preference of *A. baccarum* on these two pests was investigated.

## Materials and Methods

### Collection and Handling of *Anystis baccarum*

Samples of *A. baccarum* were collected from several locations in the vicinity of České Budějovice, Czech Republic, primarily in a home garden using an entomological beating sheet and a mouth aspirator. Mites were collected from several species of plants including peppermint (*Mentha piperita* L.), oregano (*Origanum vulgare* L.), common sage (*Salvia officinalis* L.), and raspberry (*Rubus idaeus* L.). A 190 ml glass bottle, with a raspberry leaf disc (diameter: 5.0 cm) placed with the abaxial surface facing upward on a moist cotton pad, was used to keep the collected *A. baccarum* until they were used for the experiments. Each bottle contained a maximum of 2 *A. baccarum* and an appropriate number of pea aphids (*Acyrthosiphon pisum* (Harris)) as food. To minimize cannibalism, a bundle of three plastic drinking straws (length: approximately 4.0 cm) tied together with a rubber band was added to provide shelter. All bottles containing *A. baccarum* were maintained at a photoperiod of 16L:8D, a thermoperiod of 25:20 °C (L:D), and approximately 70% RH. All experiments took place within 5 d of collection. Owing to the difficulty in establishing *A. baccarum* in the laboratory, field-collected *A. baccarum* were used directly for the functional response experiments. All individuals of *A. baccarum* selected for the experiments had a length of approximately 1.0 mm, consistent with adult size (Meyer and Ueckermann 1987).

### Confirmation of the *Anystis* Species

A total of 94 *Anystis* individuals were used in all experiments. Fifty-six were preserved for identification, of which 41 were slide mounted for morphology-based identification by us, 15 samples were sent to Fred Beaulieu and Wayne Knee (Agriculture & Agri-Food Canada, Ottawa, Canada) for species confirmation using morphology and molecular-based tools.

For morphological identification in the Czech Republic, *Anystis* mites were cleared for at least 18 h in 80% lactic acid (Lach-Ner, Czech Republic) at room temperature. The mite was then placed in a fresh drop of lactic acid on a microscopic slide. Four approximately 2-mm-long copper wires (diameter: 0.1 mm) were placed on the four sides of the mite in a drop of lactic acid, and a cover glass was added. The copper wires were used to support the cover glass so that the mite was not crushed. The microscope slide was heated on a hot plate for at least 2 min at 80 °C. The heating period was extended if necessary, depending on the clearing process. Morphological identification was performed with reference to Meyer and Ueckermann (1987) and Cuthbertson et al. (2014).

### Laboratory Rearing of *Aphis idaei* and *Neotetranychus rubi*

The culture of *A. idaei* was established on potted red raspberry plants (cv. ‘Tulameen’, tissue culture origin) placed in an insect cage (40 × 40 × 40 cm) with fine mesh and kept in a greenhouse. The greenhouse was under natural light conditions during the summer, and artificial lighting with metal-halide lamps to ensure a photoperiod of 16L:8D for the remainder of the year. The temperature was maintained through passive ventilation using automated air vents throughout the year.

Similarly, *N. rubi* was also established on potted red raspberry plants (cv. ‘Tulameen’, tissue culture origin) but these were maintained in a climate-controlled chamber with a photoperiod of 16L:8D, a temperature of approximately 24 °C, and approximately 50% RH.

### Functional Response Experiments

The experimental arena was designed with a 30 ml polypropylene storage sample container with a screw cap (Kartell S.p.A—Labware Division, Noviglio, Italy). Three holes (diameter: approximately 0.2 cm) were drilled in the screw cap, and a fine mesh was placed over them for ventilation. A freshly excised raspberry leaf disc (diameter: 4.0 cm) was placed with the abaxial surface facing upward on a moist filter paper (diameter: 4.5 cm) in the arena. All raspberry leaves used in this study were from potted wild raspberry plants of tissue culture origin. As an extra precaution to prevent *A. baccarum* from escaping, a thin strip (width: 1.0 cm) of Fluon GPI (Polytetrafluoroethylene, PTFE, Whitford Plastics Ltd., Cheshire, England) was applied near the opening of the container. Before the experiment, the *A. baccarum* were starved for 24 h by placing them individually in a 25 ml rolled rim clear glass vials (diameter × height: 2.5 × 6.0 cm) closed with fine mesh. During the starvation period, bottles were placed in a climate-controlled cabinet with a photoperiod of 16L:8D, at a thermoperiod of 25:20 °C (L:D), and approximately 70% RH.

Six densities of *A. idaei* nymphs, second or third instars, (2, 4, 8, 12, 16, and 24) and adult *N. rubi* females (2, 4, 8, 16, 24, and 32) were used for the respective functional response experiments. In the case of *A. idaei*, the nymphs were transferred using a minuten pin (diameter: 0.20 mm) to the prepared leaf disc approximately 24 h earlier to allow them to settle. However, *N. rubi* were transferred using a minuten pin or a custom-made sucking apparatus to the prepared leaf disc only approximately 1 h prior to the experiment to reduce the number of eggs laid by *N. rubi* during this period. A starved *A. baccarum* was released into each of the prepared densities to begin the experiment, and the edges of all experimental arenas were sealed with parafilm after the screw cap was closed as a precaution to prevent any escape of prey or predator. The experiment lasted 24 h, and the number of killed prey was recorded at the end of the experiment. No consumed prey was replaced during the experiment. The experiment was repeated 10 times for each prey density and for each prey species. The functional response experiment was conducted at a thermoperiod of 25:20 °C (L:D), a 16L:8D photoperiod, and a humidity of approximately 70% RH. After the experiment, *A. baccarum* was fed *A. pisum* for at least 24 h and then starved again before being used for another prey species and subsequent prey preference experiment. Each *A. baccarum* was not reused for any functional response experiment involving the same prey species.

### Prey Preference Experiments

This experiment aimed to investigate the prey preference of *A. baccarum* when both *A. idaei* and *N. rubi* were present simultaneously. All preparations and experimental design were the same as those described in the functional response experiment, except for the number of prey used. Five individuals of second or third instars of *A. idaei* and five adult females of *N. rubi* were transferred to a prepared raspberry leaf disc in the same experimental arena. There were 30 replicates for this experiment. The experiment was carried out at 25:20 °C (L:D), a photoperiod of 16L:8D, and a humidity of approximately 70% RH.

### Statistical Analysis

All statistical analyses were performed in R, version 4.4.1 (The R Foundation, https://www.r-project.org/). The functional response analysis was carried out using the *frair* package. Detailed description, explanation, and R coding required to run the package are provided in Pritchard et al. (2017). In general, the functional response analysis of this study involved 2 steps: (i) model selection and (ii) model fitting. In this study, model selection was first performed using the *frair_test* function. To fit the model and confirm output of the selection using the *frair_test* function, two models were fitted using the *frair_fit* function, with the parameter *response* specified as ‘rogersII’ for Roger’s Type II response, or ‘flexpnr’ with a scaling exponent parameter (*q*) set to *q ≠ *0, which represents a Type III response or a flexible model without prey replacement. The two models were compared using the Akaike Information Criterion (AIC), in which the model with the lowest AIC score indicates a better fitted model. Once the model was confirmed with AIC and fitted with *frair_fit*, the capture rate, *ɑ*, and handling time, *T*_*h*_, of the model could be viewed using the *print ()* function. Finally, before the graph was plotted, the *frair_boot* and *confint* functions were used to obtain the limits of the confidence intervals (CIs) of the capture rate and handling time based on 999 bootstrap replicates. The CIs were estimated using Bias-Corrected and Accelerated (BCa) method. The graph was then plotted with the help of the *drawpoly* function embedded as part of the *frair* package. Lastly, the parameters of the models for each prey species, namely capture rate and handling time, were compared using the *frair_compare (model_1, model_2)* function, where *model_1* and *model_2* were replaced with the names assigned to the fitted models.

Statistical analysis of the prey preference of *A. baccarum* was carried out using binomial generalized linear mixed model (GLMM) with the *lme4* package (Bates et al. 2015). The model was fitted using the *glmer* function, with the probability of killed prey as the response variable, prey type (eg *A. idaei* or *N. rubi*) as the fixed effect, and leaf disc as the random effect. The backward elimination method was applied using the *drop1* function with Chi-square (χ^2^) as the test statistic. The model with fixed effect (ie prey type) was found to be significantly better than the null model (χ^2^, *P *< 0.05), and was therefore selected for the analysis. The *summary ()* function was used to display the output of the binomial GLMM.

## Results

### Identification of *Anystis baccarum*

**Fig. 1.**
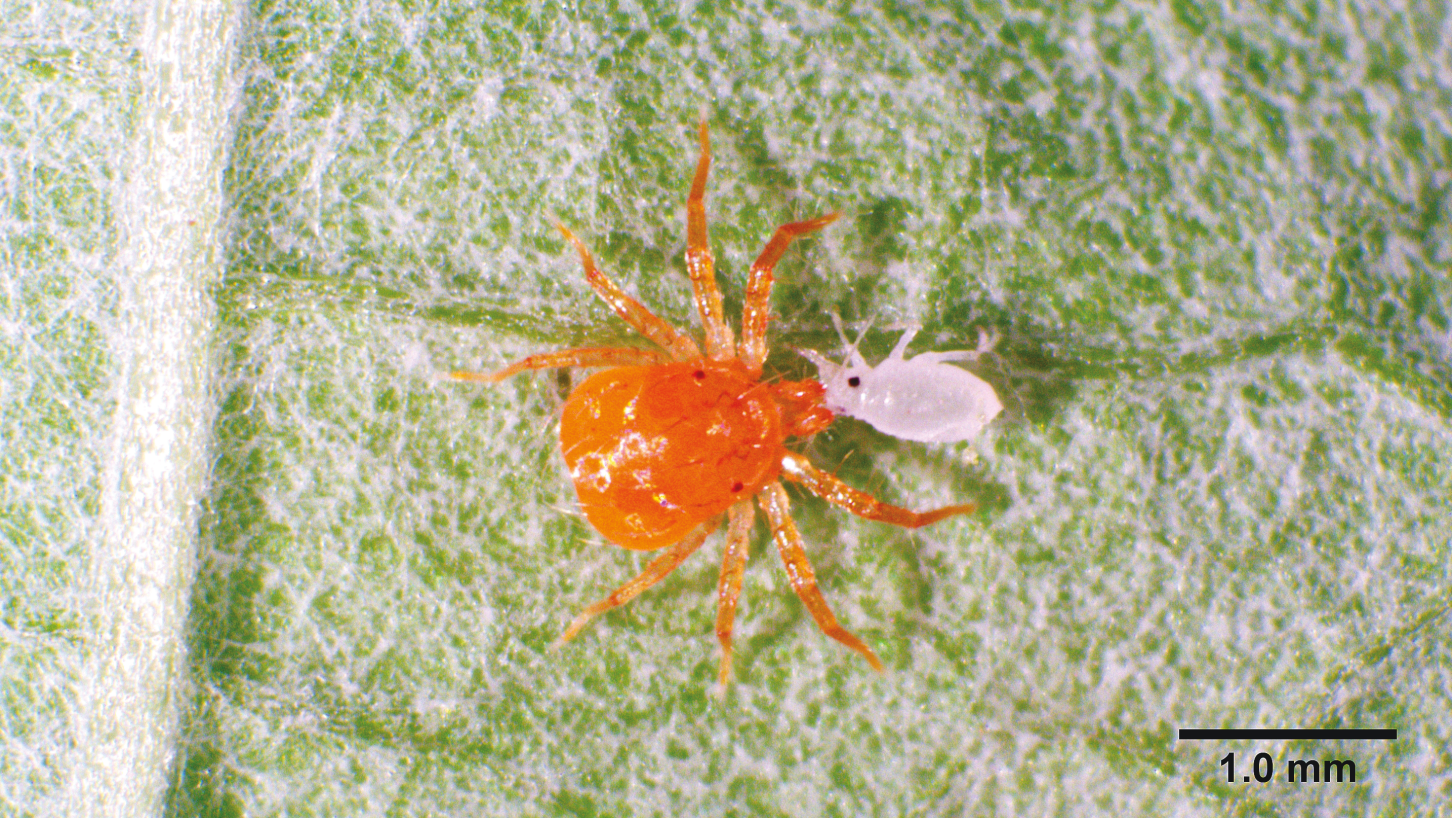
Female *A. baccarum* feeding on the nymph of the small raspberry aphid, *A. idaei*, on a red raspberry leaflet.

Both morphology and molecular identification confirmed that all preserved *Anystis* specimens were *A. baccarum* (Fig. 1). Furthermore, all 41 morphologically identified *A. baccarum* were female adults. This suggests that it is reasonable to conclude that all field-collected *A. baccarum* of even size, approximately 1.0 mm in length, used in this study were most likely female adults, as males have not been documented in this species.

### Functional Response of *Anystis baccarum*

**Fig. 2.**
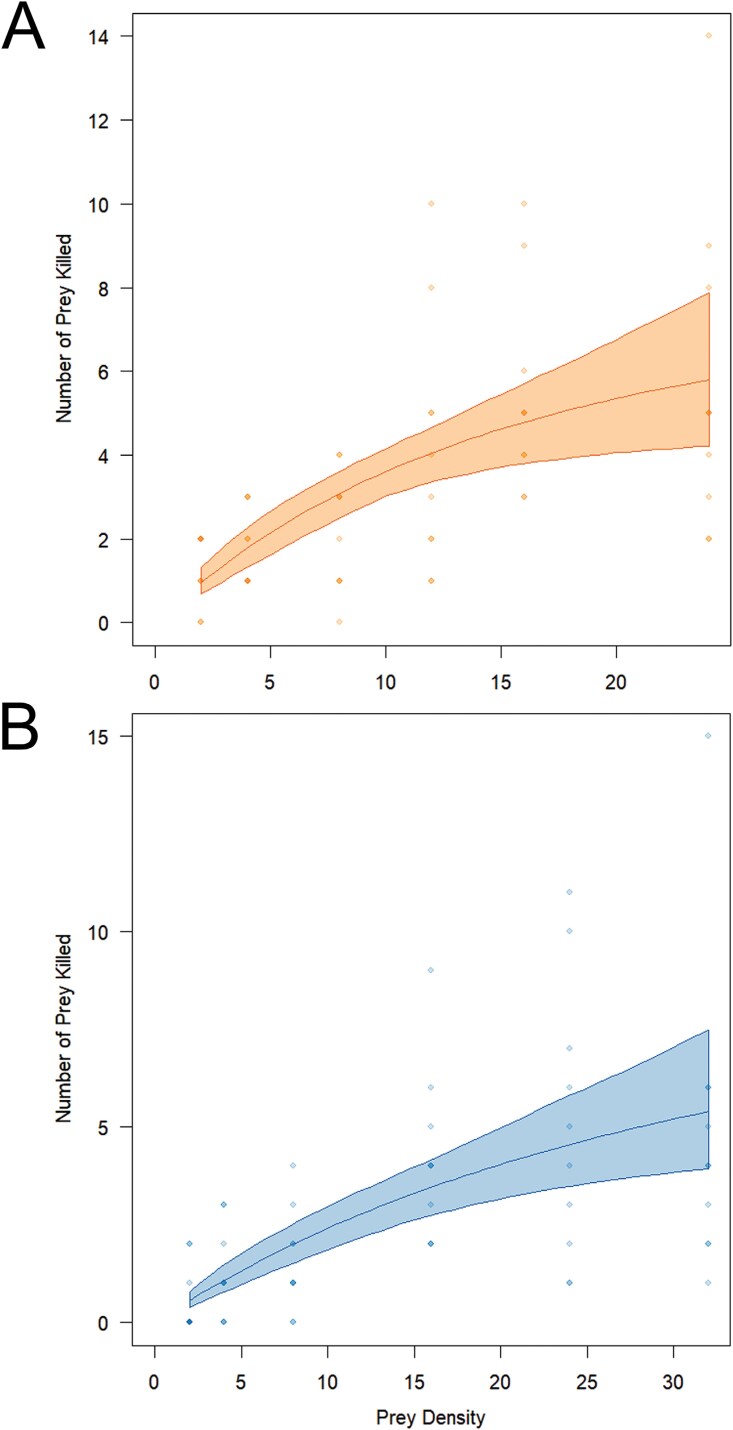
Functional response of *A. baccarum* to (A) *A. idaei* and (B) *N. rubi*. The solid lines show the type-II functional response curve fitted by the Rogers’ random predator (RRP) equation, and the shaded areas surrounding them represent the limits of the confidence interval based on 999 bootstrap replicates. The dots show the empirical data of the experiments.

The highest mean number of *A. idaei* killed by field-collected *A. baccarum* was 5.7 (SE = 1.17) at the highest test density of 24 individuals. The highest mean number of *N. rubi* killed was 5.0 (SE = 1.12) at the second highest test density of 24 individuals (Table 1). The predatory mite displayed type II functional response to both prey (Fig. 2). The curves of both functional response graphs did not show any drastic steepness but instead a rather smooth increase toward higher density, and did not reach a plateau, even at the highest prey density tested in this study. However, based on the fitted curves (Fig. 2), the number of prey killed by *A. baccarum* slowed with the increasing prey density, suggesting that it could eventually reach a saturation of predation. Based on the model parameters, *A. baccarum* had a higher capture rate (*ɑ* = 0.7295 h^-1^, SE = 0.16) when preying on *A. idaei* compared to *N. rubi* (*ɑ *=* *0.3509 h^-1^, SE = 0.35) (Table 2). In contrast, the handling time for *A. idaei* (*T*_*h*_* *= 0.1069 h, SE = 0.03) was slightly longer compared to *N. rubi* (*T*_*h*_*=* 0.0874 h, SE = 0.03). However, the statistical comparison of these fitted parameters only found a significant difference in the capture rate (*D*ɑ** = 0.3783, *z *= 2.1521, *P*(*z*) = 0.03134*), and not the handling time (*Dh* = 0.0195, *z *= 0.4582, *P*(*z*) = 0.6468). The raw data are available in Supplementary Table S1 for re-running the functional response analysis in R.

**Table 1.** The mean number of prey killed by *A. baccarum* over a 24-h period, based on 10 replicates

*Aphis idaei*		*Neotetranychus rubi*	
Density	Mean ± SE	Density	Mean ± SE
2	1.3 ± 0.26	2	0.5 ± 0.27
4	1.8 ± 0.29	4	1.2 ± 0.36
8	2.2 ± 0.44	8	1.5 ± 0.40
12	4.1 ± 0.95	16	4.1 ± 0.69
16	5.4 ± 0.75	24	5.0 ± 1.12
24	5.7 ± 1.17	32	4.8 ± 1.25

**Table 2.** Maximum likelihood estimation and confidence intervals generated from the fitted model using embedded function in the *frair* package

*Aphis idaei*						
	Maximum likelihood estimate				Confidence limits^b^	
Coefficient^a^	Estimate	SE	*Z*	*P*(z)	Lower	Upper
*ɑ*	0.729526	0.160454	4.5466	<0.001	0.421	1.367
*T*_*h*_	0.106875	0.027574	3.8760	<0.001	0.006	0.188

*Neotetranychus rubi*						
	Maximum likelihood estimate				Confidence limits	
Coefficient	Estimate	SE	Z	P(z)	Lower	Upper
*ɑ*	0.350919	0.072841	4.8176	<0.001	0.224	0.600
*T*_*h*_	0.087423	0.032351	2.7023	<0.001	0.000	0.174

^a^ *ɑ* represents the capture rate, *T*_*h*_ represents the handling time.

^b^ The confidence interval limits were obtained through 999 bootstrap replicates and estimated using Bias-Corrected and Accelerated (BCa) method.

### Prey Preference of *Anystis baccarum*

The GLMM analysis indicated that *N. rubi* was less likely to be consumed by *A. baccarum* than was *A. idaei* when both prey were present on the same leaf disc (Table 3). The fitted model has a deviance of 153.3 and 57 residual degrees of freedom. The raw data are available in Supplementary Table S2 for re-running the GLMMs analysis in R.

**Table 3.** The GLMM analysis of the prey preference of *A. baccarum* between the 2 prey

Parameter	ꞵ coefficient	SE	Z value	*P*-value (z)	Odds ratio (%)
Intercept^a^	−0.5769	0.2225	−2.592	0.00953	
*N. rubi*	−1.8934	0.3480	−5.442	<0.001	0.15056 (15.1%)

^a^Intercept is the reference level of the model

## Discussion

This study is the first to investigate the potential of *A. baccarum* as a biological control agent against raspberry pests, namely aphids and phytophagous mites, following the discovery of its natural presence on red raspberry in Norway (Tan et al. 2024b). Based on the hyperbolic shape of the graphs shown in Fig. 2, *A. baccarum* exhibited type II functional response to both pests, *A. idaei* and *N. rubi* (Holling 1959). In addition, the functional response were fitted by Rogers’ random predator equation to account for prey depletion since the study was carried out without prey replacement (Rogers 1972). *Anystis baccarum* also displayed a Holling type II functional response to the smaller green leafhopper, *E. vitis,* in tea (Zeng et al. 2007) and *Tetranychus turkestani* (Ugarov & Nikolskii) in cowpea (Khanjani et al. 1999). The type II functional response is also common among many phytoseiid mites (Fathipour and Maleknia 2016, Bazgir et al. 2020), and insect predators and parasitoids (Fernández-arhex and Corley 2003, Pehlivan et al. 2020, Wu et al. 2023, Yiacoumi et al. 2024). The type II or III functional response is a desirable trait for predators to be considered promising biological control agents, as they are characterized by an increasing attack rate on targets in the initial phase of the graph, until it reaches an asymptote, which represents the handling capacity of the predator (Holling 1959, Cicero et al. 2024, Yiacoumi et al. 2024). Therefore, in general, *A. baccarum* can be considered a suitable biocontrol agent for the management of *A. idaei* and *N. rubi*. However, the capture rate was significantly higher for *A. idaei* indicating that *A. baccarum* was more efficient against *A. idaei* (*ɑ* = 0.7295 h^-1^, SE = 0.16) than against *N. rubi* (*ɑ* = 0.3509 h^-1^, SE = 0.35). Although the handling time for *N. rubi* (*T*_*h*_* *= 0.0874 h, SE = 0.03) was slightly lower than that for *A. idaei* (*T*_*h*_* *= 0.1069 h, SE = 0.03), there was no statistically significant difference, and the mean number of *A. idaei* killed was also higher. Therefore, *A. baccarum* may be a better biocontrol agent for *A. idaei*. In our study, neither of the fitted curve (Fig. 2) increased dramatically at low prey densities, suggesting that *A. baccarum* did not feed vigorously on the prey during the 24 h. This may be related to the biological stages of the adult predatory mites, in which adult phytoseiid mites, such as *Phytoseius plumifer* (Canestrini & Fanzago) and *Neoseiulus barkeri* Hughes, were observed to have higher predation rates during the preoviposition and oviposition periods than during the postoviposition period (Al-Azzazy 2021, Al-Azzazy and Alhewairini 2024). Although this behavior has not been studied in *A. baccarum*, there is still the possibility that it behaves similarly. Therefore, in the field-collected individuals, there were most likely adults in different biological stages, resulting in different predation rates. This can cause greater variability in the empirical data, resulting in fitted curves that are less steep at lower prey densities, particularly because *A. baccarum* deutonymphs present a higher and more consistent predation rate, even at lower densities of *E. vitis* (Zeng et al. 2007), even though this prey is larger than both *A. idaei* and *N. rubi*. However, since *A. baccarum* has been reported to be naturally present on raspberry (Tan et al. 2024b), the results of the present study provide valuable information on adult *A. baccarum* as a conservation biocontrol agent, as field-collected individuals can better represent natural conditions. The success of conservation biocontrol can also reduce reliance on commercial products and lower the cost of crop protection. Future studies using different stages of life of *A. baccarum*, specifically deutonymphs and tritonymphs, could be carried out for comparison with field-collected adults.

The mean number of *A. idaei* killed by *A. baccarum* within 24 h ranged from 5 to 6 individuals and the mean number of *N. rubi* killed by *A. baccarum* was approximately five individuals. Field-collected adult *A. baccarum* were previously reported to consume an average of 6.2 *P. ulmi* and 5.2 *Bryobia rubrioculus* (Scheuten) per day on apple leaf discs (Cuthbertson and Murchie 2004), and comparable to our study. However, although laboratory-reared *A. baccarum* consumed an average of 6.1 and 7.8 first-instar foxglove aphids, *A. solani*, on pansy leaves in an experiment (Saito and Brownbridge 2021), field-collected *A. baccarum* consumed an average of only 1.2 final nymphal instar *R. insertum* on apple leaf discs within 24 h (Cuthbertson and Murchie 2004). The difference may be due to several factors, but size was probably one of them, as it can affect the handling capacity of the predator (Aljetlawi et al. 2004). For example, *R. insertum* was in an older nymph stage and was larger in size (Hullé et al. 2020). The effect of prey size on the feeding rate was demonstrated by Saito and Brownbridge (2021), where the mean number of adult *A. solani* consumed was not significantly different between the control (absence of natural enemies) and treatment with the release of *A. baccarum*, but the mean number of nymphs consumed was significant. Although it cannot be directly compared, based on the average number of aphids consumed, laboratory-reared *A. baccarum* does not seem to have a very different feeding rate than the field-collected *A. baccarum* from this study. Predation by *A. baccarum* has been reported to be improved in the control of phytophagous mites on smooth leaf surfaces (Gerson 1992, Cuthbertson and Murchie 2004). However, in this study, there was no obvious negative effect of the “hairy” abaxial red raspberry leaf surface on the feeding rate of *A. baccarum*.

In non-cultivated raspberry plots with dense vegetation, *A. baccarum* has been hypothesized to feed more frequently on alternative food sources than on phytophagous mites (Tan et al. 2024b). The results on the prey preference of *A. baccarum* demonstrated that, between *N. rubi* and *A. idaei*, the latter was more likely to be consumed by *A. baccarum*. This may be particularly related to the searching behavior of *A. baccarum*, which is described as chance-dependent (Gerson et al. 2003), and *A. idaei*, being a larger prey, may be easier to find. Furthermore, the capture rate estimated from the functional response also indicated that *A. baccarum* was more effective at capturing *A. idaei* than *N. rubi*. This behavior of *A. baccarum* may also represent an intentional diet selection, as noted in *Anystis agilis* (Banks) (Sorensen et al. 1976). *Anystis agilis* loses its bright orange coloration and behaves more sluggishly and uncoordinated under laboratory conditions when *T. urticae* is the only food source. However, more in-depth studies must be carried out to investigate the biology of *A. baccarum* in relation to various diets, including pollen. In addition to their probable diet selection, the webbing and silk nests of spider mites can impede the predation effectiveness of *Anystis* mites, likely due to their large bodies, which make it difficult to maneuver through the silk and webbing (Sorensen et al. 1976, Ito 2020). This characteristic, along with its cannibalistic nature and long generation time, makes relying solely on *Anystis* species as biocontrol agents unsuitable. However, synergistic interactions between *A. baccarum* and other biocontrol agents, particularly those that complement the characteristics of *A. baccarum*, have been reported, despite these interactions sometimes being affected by intraguild predation by *A. baccarum*. Such synergistic interactions have been observed in several combinations under laboratory conditions, for example, *A. baccarum* in combination with *Neoseiulus cucumeris* (Oudemans) to suppress *T. urticae* and *F. occidentalis*, with the aphid parasitoid *Aphidius ervi* Haliday to control *A. solani*, and with the parasitoid *Dolichogenidea tasmanica* (Cameron) to manage populations of *Epiphyas postvittana* (Walker) (Paull et al. 2012, Saito and Brownbridge 2022, Saito et al. 2023). In the field, these interactions may be difficult to monitor, but such functional compatibility can be observed from the densities of prey, such as the better suppression of *P. gracilis* and spider mite densities when *N. cucumeris, Typhlodromus* (*Typhlodromus*) *pyri* Scheuten, and *A. baccarum* are present in raspberry (Tan et al. 2024b). However, more studies will be necessary to better understand the additive or synergistic interactions between biocontrol agents in controlling various raspberry pests.

In conclusion, to our knowledge, this is the first study on the functional responses of *A. baccarum* to raspberry pests. *Anystis baccarum* was not previously documented on raspberry until Tan et al. (2024b). Therefore, the findings of this study provide valuable insight into the potential of *A. baccarum* as a biocontrol agent for raspberry. Despite previous studies reporting it as an important generalist predatory mite in orchards and a cosmopolitan species present on a wide range of crops, feeding on a variety of prey, it has not received as much attention compared to more commonly known phytoseiid predatory mites (Cuthbertson et al. 2014, Saito et al. 2023). More importantly, it has the potential to be promoted as a conservation biocontrol agent (Cuthbertson and Murchie 2010), which can help farmers to reduce reliance on commercial products and possibly lower production costs. These findings can serve as an important reference for future studies on the management of other economically important pests using *A. baccarum*, especially thrips, which are emerging pests in raspberry (Tan et al. 2024a). However, much more needs to be understood about this predatory mite before *A. baccarum* can be reliably incorporated into comprehensive pest management strategy, especially its interactions with other biological control agents in raspberry crops. Further studies should also investigate the nutritional ecology of *A. baccarum* in relation to different diets, such as pollen, as this will affect the effectiveness of biological control approaches. Despite the knowledge gaps surrounding *A. baccarum*, raspberry growers now have an additional option for sustainable biological control of raspberry pests, and future research could lead to broader applications of this mite in other crops, contributing to more sustainable pest management in agriculture.

## Supplementary material

Supplementary material is available at *Journal of Economic Entomology* online.

toaf112_suppl_Supplementary_Material
